# Doctor-patient communication with people with intellectual disability - a qualitative study

**DOI:** 10.1186/1471-2296-10-82

**Published:** 2009-12-17

**Authors:** Magda Wullink, Wemke Veldhuijzen, Henny MJ van Schrojenstein Lantman - de Valk, Job FM Metsemakers, Geert-Jan Dinant

**Affiliations:** 1 Department of General Practice, School for Public Health and Primary Care (CAPHRI) Maastricht University, PO Box 616, 6200 MD Maastricht, the Netherlands

## Abstract

**Background:**

People with intellectual disability (ID) expressed dissatisfaction with doctor-patient communication and mentioned certain preferences for this communication (our research). Since many people with ID in the Netherlands have recently moved from residential care facilities to supported accommodations in the community, medical care for them was transferred from ID physicians (IDPs) to general practitioners (GPs) in the vicinity of the new accommodation. We addressed the following research question: 'What are the similarities and differences between the communication preferences of people with ID and the professional criteria for doctor-patient communication by GPs?'

**Methods:**

A focus group meeting and interviews were used to identify the preferences of 12 persons with ID for good communication with their GP; these were compared with communication criteria used to assess trainee GPs, as described in the MAAS-Global manual.

**Results:**

Eight preferences for doctor-patient communication were formulated by the people with ID. Six of them matched the criteria used for GPs. Improvements are required as regards the time available for consultation, demonstrating physical examinations before applying them and triadic communication.

**Conclusions:**

People with ID hold strong views on communication with their doctors during consultations. GPs, people with ID and their support workers can further fine-tune their communication skills.

## Background

In the last decade, thousands of people with intellectual disability (ID) in the Netherlands have moved from residential care facilities to supported accommodations in the community [1]. These are mainly persons with mild to moderate levels of ID, but people with profound to severe ID are now also living in the community [2]. Consequently, the medical care for these people has been transferred from the general practitioner (GP) or ID physician (IDP) connected to the residential care facility to local GPs in the neighbourhood of the new accommodations. Before, most GPs had 10-12 registered patients with ID in their practice [3], but this number is now rising. GPs are the gate keeper in the Dutch health care system. By including individualisation and participation in the community as the guiding principles in care provision, GPs are expected to take on more responsibility for the health of people with ID, but are unprepared for this increasing task.

People with ID frequently complain about the communication with doctors during consultations [4,5]. Persons with ID who participated in our studies told us that they often felt that GPs did not understand them. This was also emphasised by people with ID who were on the client panel of the 'Innovation of health care for people with ID' research programme of the Netherlands Organisation for Health Research and Development (ZonMw; personal information).

In one study, 78% of people with ID had visited their physician during the previous year [6], while Straetmans [7] found an average of 5.4 contacts of people with ID with their GP in one year, compared to 3.2 contacts for people without ID. Because of the higher frequency of visits and because of their dissatisfaction, it is understandable that people with ID have preferences concerning doctor-patient communication. Doctor-patient communication may also be hampered by the GPs' lack of awareness of visual impairment and hearing loss among people with ID, which are frequently underdiagnosed [8-10].

During their traineeship, GPs are trained to communicate on the basis of professional criteria. During our work with GPs to establish a Transfer of Care Guideline [11], and during the development of an individual post-graduate education programme for ID care [12], we found that GPs did not want a paragraph about communication criteria for patients with ID to be included in these documents. Their opinion was that they had been very thoroughly trained in communication and that their professional communication criteria sufficed for the communication with people with ID.

There was thus an obvious gap between the people with ID, who felt that doctor-patient communication should be improved, and GPs who believed that their professional communication criteria skills were sufficient to meet the needs of people with ID. The present study tried to bridge this gap. The study design included a focus group session and semi-structured interviews. The study aimed to explore to what extent professional communication criteria for GPs correspond with the needs of people with ID and what additional requirements might be necessary. The research question was: 'What are the similarities and differences between communication preferences of people with ID and the professional criteria used to assess doctor-patient communication by GPs?'

The definition of intellectual disability of Luckasson [13] was used; this definition states that ID is characterized by significant limitations in intellectual functioning and in adaptive behaviour as expressed in conceptual, practical and social adaptive skills; the disability originates before age 18.

## Methods

### Data collection

As part of the Transfer of Care Guideline study, intended to facilitate the transfer of medical care from IDPs to GPs [11], we organised a focus group discussion and interviewed people with ID. During the focus group meeting and two interviews, the participants worked together with the researcher (MW) to formulate their communication preferences.

As participants for our focus group we recruited people with ID from the client council of a residential care facility in the south-east of the Netherlands. This ensured that our participants were both interested in and able to discuss issues related to the quality of their health care. All ten council members participated, some of them living in the residential care facility, others in houses in the community. The participants had been members of the client council for over a year, and had learned to discuss all kinds of subjects with each other. The council's support worker helped ensure that all members took part in the discussion and listened to each other, thus creating a safe environment for group discussion. The regular chair of the council, a woman with ID, chaired the meeting, and the support worker was present during the focus group discussion. Before the meeting, all participants were sent written information in easy-to-understand language about the purpose and topic of the meeting. The focus group session was moderated by HvSLdV; as an IDP, she has several decades of experience in communication with people with ID. The first author was present as an observer. The goal of the meeting was to identify the communication preferences of the participants. During the focus group meeting, the observer kept notes about the content and course of the discussion. Audiotaping of this meeting was not allowed because of the vulnerability of the participants. The chair, the support worker and the moderator assisted in the process of discussion, to ensure that all members of the client council could participate.

For the purpose of this study, we adjusted focus group procedures to suit the specific characteristics of our participants. The main aim was to support our participants in accurately formulating their preferences for the communication with GPs. At the start of the discussion, we used a critical incident technique by asking the participants to describe positive and negative experiences in their communications with GPs. The participants were stimulated to reflect on the kind of communicative behaviour they had liked or, in the case of a negative incident, to suggest more preferable behaviour. After an incident or topic had been discussed in detail, the moderator summarised the main points of the discussion and the preferences for GP-patient communication that had been formulated. The participants were then asked whether they agreed or disagreed with the way the preference had been worded, or whether they would prefer to alter it. At the end of the focus group meeting, the researcher read out all formulated preferences and again asked for confirmation (participant check). This 'on the spot' participant check was chosen because, in our experience, people with ID appreciate discussions and make suggestions during group meetings or individual interviews, but checking the accuracy of interpretations with them on a later occasion is difficult.

To triangulate the data from the focus group and to widen the sample of participants, two semi-structured interviews were held in another part of the country. Two client council members from the ZonMw research consultation group were recruited: one member from the north-west of the country and one from the south-west of the country. The first interview was carried out by telephone; the second was a face-to-face interview in the presence of the participant's support worker. Before the interviews, written information in easy-to-understand language was sent to the interviewees. The interviewer took notes during the interviews. The methodology was similar to that used in the focus group interviews. Participants were first asked to reflect on their experiences and stimulated to formulate preferences for GP behaviour. These preferences and those formulated in the focus group were then submitted to the interviewees and they were asked to confirm or reject these preferences or to suggest alterations. The resulting list of preferences, which had been confirmed by the focus group participants and further validated and expanded in the individual interviews, was considered the final list and used for further analysis.

The Maastricht University Hospital/Maastricht University Medical Ethics Committee gave its consent to this qualitative study proposal. People with ID and their relatives or legal representatives were asked to fill in and sign an informed consent form before participating in interviews.

### Analysis

The preferences of people with ID concerning communication with their GPs, as formulated during the focus group sessions and interviews, were then compared with the communication criteria for GPs included in the MAAS-Global manual [14]. The MAAS-Global is an assessment instrument for communication skills which can be compared to the Calgary-Cambridge Guides [15]. The MAAS-Global is also the most widely used guideline for GP-patient communication in general practice training in the Netherlands [16]. The instrument is accompanied by a manual listing the criteria for 'excellent' communication skills (SE Appendix: Addendum - introduction, follow-up consultation, request for help, physical examination, diagnosis, management, evaluation of consultation, exploration, emotions, information provision, summarising, structuring and empathy). The wording of the preferences formulated by people with ID and the criteria in the manual were analysed (content analysis) [17]. For each of the items in the MAAS-Global manual, the assessors scored whether it matched one or more of the preferences expressed by the people with ID. Similarities and differences with these preferences were coded. The content analysis was conducted by the first author (MW) and an independent researcher, who discussed any differences of interpretation until consensus was reached. The researchers underlined the similarities in the MAAS-Global manual, and the number of the corresponding preference was written against it in the margin (initial agreement). The overall degree of agreement was calculated by counting the difference and similarity scores for each preference, calculating the percentage of similarity for each preference and calculating the mean percentage of similarity with the preferences.

## Results

### Participants

All members of the client council (seven women and three men) participated in the focus group discussion. They were all middle-aged, had mild/moderate levels of ID and could speak for themselves (i.e. were verbally competent). All members would normally visit the doctor together with their support worker.

Both interviewees (one woman and one man) were verbally competent, had a mild level of ID and were middle-aged. Interviewee 1 explained that she usually visited her GP on her own, while the second interviewee visited his doctor together with his support worker.

### Focus group meeting and interviews

At the client council's initiative, they discussed the information they had received with the council's support worker before the focus group meeting. During the meeting, participants often interrupted each other, but the chair, the support worker and the moderator assisted the process of discussion and ensured that all participants could finish their stories and tell what they wanted to tell. Although the council members and the interviewees visited different doctors and had different experiences of doctor-patient communication, this did not prevent them from reaching agreement about preferences. The preferences they formulated were based on both positive and negative experiences with doctor-patient communication.

### Preferences

The people with ID held strong views on doctor-patient communication. During the focus group discussion and the interviews, clients made statements like:

*'My doctor did not talk to me, but to my support worker' *(focus group member).

*'My doctor did not ask permission to talk to my support worker about me' *(interviewee 2).

*'I go to the doctor without my support worker. I can talk very well with him' *(interviewee 1).

The participants formulated the following preferences for communication with their doctors:

*1. The doctor should allow me to tell him/her about my symptoms*.

*2. The doctor should ask me questions about my symptoms*.

*3. The doctor should listen carefully to me*.

*4. The doctor should take me seriously*.

*5. The doctor should take sufficient time for the practice visit*.

*6. The doctor should show consideration for what I want*.

*7. The doctor should explain and demonstrate before starting a physical examination*.

*8. The doctor should ask me for permission before talking to my support worker about me*.

Preferences 1-7 were formulated during the focus group meeting. Interviewee 1 suggested eight preferences for communication with her GP, adding one new preference (no. 8) to the seven formulated by the focus group. Preference 8 was also the first one to be expressed by interviewee 2 and considered by him to be the most important one. In addition, he spontaneously mentioned the seven other preferences that had been formulated by the focus group. During our study, all participants told the researcher that they appreciated being asked to participate.

### Comparison of preferences and criteria

Comparing the preferences expressed by people with ID with the criteria listed in the MAAS-Global manual was complicated by that fact that the preferences were a combination of behaviours and attitudes, whereas the manual only describes behaviour. In addition, the people with ID and the GPs differ in their presuppositions and the language they used. This initially resulted in a low level of agreement between the two researchers who did the content analysis (55%). After some discussion, however, consensus was usually easily reached. Preferences 3 (listening), 5 (time) and 6 (showing consideration) led to the most discussion. Although listening was not explicitly described in the manual, doctors behaved in accordance with other relevant criteria, e.g. checking if requests for help have been addressed, giving patients room to respond and summarising. Preference 5 was discussed extensively. The people with ID wanted to have more time to talk to the doctor. They felt there was too little time for communication during a regular consultation; the normal scheduled time is 10 minutes, and occasionally GPs had planned double consultation times for patients with ID. The discussion considered the feeling of insufficient time that was expressed by the patients with ID and the criterion for sufficient consultation time contained in the Maas-Global manual. During the discussion, one of the authors of the Maas-Global manual was consulted by e-mail. He stated that the manual did make recommendations for consultation time, but that in practice, consultation time is set at 10 minutes, based remuneration agreements between health care funding bodies and GPs. We therefore decided to omit this item from Table 1, which presents the agreement between preferences and criteria. The discussion on preference 6 related to showing consideration. The manual did mention certain aspects of consideration, e.g. checking patient's questions, anticipating patient's reactions to the examination, asking patient's reaction and discussing the management strategy with the patient with ID and with the support worker.

**Table 1:** Correspondence between communication preferences by people with ID and criteria used in GP training*

Preferences: Criteria:	1: telling symptoms	2: asking questions	3: listening	4: taking seriously	5: time	6: showing consideration	7: demonstrating physical examination	8: talking to support worker
Item 1 Introduction	X	X						
Item 2 Follow-up consultation		X						
Item 3 Request for help	X	X	X			X		
Item 4 Physical examination				X		X	X	
Item 5 Diagnosis		X		X		X		
Item 6 Management				X		X		
Item 7 Evaluation of consultation			X	X		X		
Item 8 Exploration	X	X	X	X		X		
Item 9 Emotions				X				
Item 10 Information provision				X				
Item 11 Summarising	X		X			X		
Item 12 Structuring								
Item 13 Empathy	X			X				

* preferences derived from focus group discussion and interviews with people with ID; criteria from MAAS-Global Manual

### Similarities and differences between criteria and preferences

Similarities with the criteria in the manual were found for preferences 1 (tell symptoms), 2 (ask questions), 3 (listening), 4 (taking seriously), 6 (showing consideration) and (partly) 7 (physical examination). Preferences 5 and 8 did not fully correspond with the criteria. In the case of preference 5 (consultation time), this was because the people with ID felt there was too little time for the consultation, and this aspect was only implicitly included in the criteria for good communication. As regards number 8 (talking to the support worker) there was a complicating factor, namely triadic communication. During consultations with a person with ID, the support worker was usually in the consultation room as well, and actively took part in the communication. The preference expressed by the people with ID was that they themselves should be the person addressed primarily by the doctor, but this appeared not to be regular practice. The communication criteria gave no suggestions about handling triadic communication.

## Discussion

In this qualitative study, we interviewed people with ID in a focus group meeting and during semi-structured individual interviews. Our aim was to explore the extent to which professional communication criteria used to assess trainee GPs correspond with the needs of people with ID, and what additional requirements could be identified.

### Strengths and weaknesses

To our knowledge, this was the first study in which persons with ID themselves were asked to state their communication preferences, as policies with regard to health care for people with ID are usually paternalistic, in the sense that they are based on assumptions of benevolent others about what people with ID need. Lennox [18] recommended not abandoning this group of people, who often experience social exclusion [6]. The participants appreciated being asked to participate in the study, and social exclusion may be an important aspect of the discussion about doctor-patient communication. GPs and support workers can improve their own communication skills and also support people with ID in improving theirs. The potential advantage of improving communication skills, both for health care workers and the people with ID themselves, is that it may help people with ID exercise autonomy and that it may lead to improved health care.

It is unclear if data saturation was achieved, as only one focus group meeting and two interviews were performed. But interviewee 1 added only one new point to the list of seven preferences established the focus group, and interviewee 2 confirmed these eight preferences, which suggests that we were at least close to saturation. In conducting this study, we were hampered by regulations intended to protect this vulnerable group of people in research, in that privacy rules prevented audiotaping or videotaping. This meant that no verbatim transcription was possible, precluding a thorough analysis of the spoken texts. As an alternative, a participant check was performed during the meeting and interviews, and the participants were asked if they could agree with the proposed wording of the preferences. We consider these limitations acceptable, as the aim of this study was to draw up a list, and not to build and test a theory.

The other requirements for qualitative focus group research were met: a homogeneous group of people with ID, a clear research question, preparation of the participants by written information in easy-to-understand language and unequivocal interpretation of the focus group and interview results, promoted by the assistance of an independent researcher.

As regards the generalisability of our results, it must be remembered that older or younger (rather than middle-aged) persons, persons with visual or hearing disabilities, persons with more severe ID or verbally incompetent persons could have other communication preferences.

### Previous research

The following recommendations on doctor-patient communication are included in the Australian Management Guidelines Developmental Disability: '... people with developmental disability appreciate doctors who: talk to them respectfully, do not shout, explain what is happening, treat them as if they are worthwhile, listen to what they are trying to say, say when they do not understand them, allow enough time for the consultation' [[19], p.17]. These issues fit in with the preferences of people with ID we found in our study, although the latter were more specific. Although there is not a great deal of literature on doctor-patient communication with regard to people with ID, there is extensive literature on doctor-patient communication in other situations [20]. For instance, triadic communication and its specific problems have been described for doctor-parent-child communication [15,21,22]. Since we found no studies on health-related triadic communication with people with ID, we refer to the studies by Tates [21,22] about doctor-parent-child communication. They state that both GP and parent have a role in educating the child in illness and health care management, so the child's participation is important. Although GPs do try to involve children into the communication during consultations, GPs and parents often do not behave in a way that is supportive to the child. When dealing with less competent patients, GPs sometimes specifically fail to ask permission to talk with someone else about the patient. The preferences of people with ID are in agreement with the recommendations formulated by Tates [21,22] for triadic communication in doctor-parent-child communication.

Another aspect of communication problems with people with ID is the underdiagnosis of visual and hearing impairments among people with ID [8-10]. These problems greatly increase the risk that people with ID may miss gestures, looks, etc. We therefore recommend direct communication by health professionals with people with ID, which was also one of the preferences expressed by the people with ID in our study.

In a study on GPs' workload and the awareness of psychological problems in patients, Zantinge [23] reported less patient-centred behaviour on the part of GPs who have 'a subjective experience of a lack of time'. Patients with ID may sense this behaviour, and this may relate to preference 5 (time) as expressed in our study. Although in other areas of professional practice good examples can be found, more emphasis should be given to the inclusion of people with ID as a reseach participant or research partner. People with ID expressed clear views in interviews; they wanted treatment on the basis of equality; they wanted to make their own decisions and wanted to have free choices [24]. People with ID in England report that for example their housing, work and payment should be improved [6]. People with ID improved their decision-making capacity with regard to sexuality issues after following a sex education intervention [25].

### Recommendations for daily practice

The people with ID in our study would appreciate improvements to the communication skills of GPs and support workers. The results of our study therefore allow the following recommendations to be formulated to improve communication. (i) Doctors should plan double consultation time to give people with ID the opportunity to formulate questions, as well as to give themselves more room so they will not feel hurried during busy practice hours. People with ID should prepare the consultation together with their support worker. (ii) Doctors should demonstrate any physical examination before starting it, becomes possible if the recommended double consultation times are used. (iii) Doctors should observe the rules of triadic communication (just as in communication with a child and a parent). Taking a training course or studying a book on communication instructions will be helpful for all parties involved in consultations. This is in agreement with an editorial about illness in people with ID by Ali & Hassiotis [26], who recommended communication skills training for health professionals. This is already established practice in Australia, where health care workers use an instruction book, the Australian Management Guidelines Developmental Disability, which was developed to support them in their contacts with people with ID [19].

The differences between the preferences expressed by our respondents and the criteria in the MAAS-Global manual and the Australian recommendations support the hypothesis that additional requirements for communication may help doctors as well as people with ID and their support workers to improve their communication skills.

### Recommendations for research, policy and practice

Research is needed to develop methods for involving people with ID in participatory research. Although our method worked well for our qualitative study to identify preferences, it would probably be insufficient for more extensive theory building.

Research is also needed into the consequences of the increased numbers of patients with ID currently being registered in general practices. The health care funding system for GPs is based on a standard practice with 2350 registered patients [27]. The influx of people with ID who have higher morbidity rates and need more consultation time because of communicative differences adds to the GPs' workload. Our study showed that people with ID need a slightly different communicative approach than that described in communication criteria for GPs. Adjustments to other aspects of GPs' usual practice may also be necessary to meet the needs of people with ID.

Another area which should be investigated is that of triadic communication between the doctor, the patient with ID and the support worker. Research should examine if the recommendations for triadic communication with children can be applied to adult people with ID; should this not be the case, then new recommendations should be formulated. In addition, the influence of visual and hearing impairment on communication during consultations should be investigated.

Recommendations for communication should be implemented by all ID health care workers as well as to people with ID. We recommend the development of training courses and an instruction book for people with ID and health care workers to improve communication.

Verbally competent people with ID can be included as research partners in communication studies. The use of videotaping to record facial expressions and gestures should be allowed for studies among verbally incompetent people with ID.

The current general communication criteria in GP training manuals should be supplemented with a section on triadic communication.

## Conclusions

Despite the differences between people with ID and GPs in terms of in presuppositions and the language they use, there are many similarities between the communication preferences of people with ID and the criteria for good doctor-patient communication specified in the MAAS-Global training manual for GPs. The most important elements that were added by our respondents with ID to the communication criteria were that doctors should demonstrate any physical examinations before carrying them out, and that doctors should address the person with ID as their principal communication partner in triadic communication. We found that it was very well possible to draw up a list of preferences of people with ID on this subject, as they held strong views on the communication with their doctors, were able to express these views and were able to confirm or modify the wording of preferences based on their views during focus group meetings.

## Competing interests

The authors declare that they have no competing interests.

## Authors' contributions

MW, HvSLdV, JM and GJD were involved in discussions that led to the original idea for the research. WV was involved in the procedures of qualitative research. MW and HvSLdV collected the data. MW was involved in analysing the data and wrote the first draft of the paper. All authors critically revised the paper for important intellectual content and approved the final version.

## Appendix

**Addendum **Criteria for 'excellent' communication skills of GPs, as described by van Thiel et al. [15]

### Communication criteria for each separate phase

Item 1 Introduction

In the initial phase of the consultation the doctor orientates himself with regard to the reason for the visit by giving the patient room to talk about his complaints, problems or questions to encourage the patient. General questions include questions about how long the patient has the problem or complaint, how serious it is and what it means to the patient. The opening question is not rated.

The doctor explores whether there are any other reasons for the patient's visit. In rating this aspect the timing of this question is crucial: before starting detailed history-taking.

Item 2 Follow-up consultation

In a follow-up consultation the doctor makes the connection with the previous consultation by naming the previous complaints, requests for help and arrangements made.

The doctor also finds out whether the patient has complied with the agreed management plan.

The doctor also asks about the course of the complaint and the effect of the treatment or management strategy.

Item 3 Request for help

The doctor names the patient's requests for help, preferences or expectations.

In addition the doctor names the reason the patient states why he came for the visit.

The doctor completes the request for help by checking whether all patient's questions, preferences or expectations have been addressed.

Item 4 Physical examination

The doctor tells the patient before he performs the physical examination where it will take place, which parts of the body should be uncovered and what the patient should do (lie, sit, etc).

The doctor explains what the examination entails and explains his further actions during the examination if necessary.

The doctor treats the patient with care and respect. He anticipates the patient's reactions to the examination, e.g. pain, and addresses them. When no physical examination is performed, either indicated or not, 'n.a.' should be circled.

When, for any reason, no physical examination is performed, n.a. should be circled.

Item 5 Diagnosis

The doctor names the main findings from the history and physical examination, followed by a diagnosis or working hypothesis.

In addition the doctor tells about the causes of the complaint or disorder, or the connection between findings and diagnosis.

The doctor gives a concrete indication to the seriousness, the expected duration of the complaint and the course, with or without treatment.

Finally, the doctor asks the patient to give his reaction to the findings, diagnosis, prognosis etc.

Item 6 Management

The doctor discusses the management strategy by letting the patient have his say by asking the patient's opinion or by making an inviting pause. The risks and benefits of the proposed management strategy are also discussed. Depending on the nature of the complaint the doctor may need to discuss alternatives or indicate that there are no alternatives. The risks and benefits of the proposed management strategy and any alternative strategies are also discussed.

The doctor talks about the feasibility of the proposed strategy taking into account the patient's possibilities and the doctor verifies if and to what extent the patient will adhere to the proposed management strategy.

The doctor makes concrete arrangements about further medical actions (who, what, when).

Finally, the doctor asks about the patient's reactions to the proposed course of action and arrangements.

Item 7 Evaluation of consultation

At the end of the consultation the doctor asks a general question about what the patient thinks or feels at this moment. The question need not concern any specific aspect of the consultation.

At the end of the consultation the doctor checks whether the patient's requests for help have been adequately addressed.

The doctor checks whether the patient has been offered perspective for the time being.

### General communication criteria

Item 8 Exploration:

The doctor explores the patient's request for help, wishes or expectations by asking questions. This should be done in an inviting manner.

The doctor explores the patient's reaction to the information given. This applies in particular to the phases 'diagnosis' and 'management'

Exploration takes place within the patient's frame of reference.

While exploring the doctor responds to nonverbal behaviour and cues.

Item 9 Emotions:

The doctor asks about the patient's feelings or he asks questions when the patient shows emotions.

The doctor reflects the feelings that the patient shows and expresses appropriately, with respect to both their nature and intensity.

The doctor pays attention to the feelings throughout the consultation by asking questions and reflecting feelings sufficiently and with an appropriate balance of time, i.e. not too much and not too little.

Item 10 Information giving

The doctor announces to the patient that he is going to give information about a subject and explains which categories will be dealt with.

The information is given in small quantities and the doctor explains details concretely.

The doctor uses language that is easy to understand for this particular patient.

The doctor checks whether the patient has understood the information by asking questions.

Item 11 Summarizations:

The doctor demonstrates throughout the consultation that he has heard what the patient has to say through sufficient and well balanced summarizations, phrases concisely, in his own words, content wise correct, and he offers the patient room to respond (pause, questioning, intonation, asking questions).

Item 12 Structuring:

The doctor gives guidance to the consultation by ordering phases in a logical way, consecutively: introduction, follow-up, consultation, request for help, history, physical examination, diagnosis, management and evaluation.

The doctor also divides his time between phases used in a well balanced way and, if necessary, intervenes to cut the story of a very talkative patient short. The doctor brings structure to the consultation by announcing the phases used.

Item 13 Empathy

The doctor's attitude is inviting and shows his concern for the patient. Also he is sincere in showing empathy. This attitude is reflected in gestures, eye contact and tone of voice.

The doctor expresses empathy in brief verbal responses.

## Pre-publication history

The pre-publication history for this paper can be accessed here:

http://www.biomedcentral.com/1471-2296/10/82/prepub
